# Physico-Chemical Characterizations of Composited Calcium-Ortho-Phosphate Porous Particles and Their Controlled Release Behavior of Clindamycin Phosphate and Amikacin Sulfate

**DOI:** 10.3390/polym16223144

**Published:** 2024-11-12

**Authors:** Namfon Khamkaew, Sorada Kanokpanont, Jirun Apinun, Chalika Wangdee, Antonella Motta, Siriporn Damrongsakkul

**Affiliations:** 1 Department of Chemical Engineering, Faculty of Engineering, Chulalongkorn University, Bangkok 10330, Thailandsiriporn.d@chula.ac.th (S.D.); 2 Center of Excellence in Biomaterial Engineering in Medical and Health, Faculty of Engineering, Chulalongkorn University, Bangkok 10330, Thailand; jirun.a@chulahospital.org (J.A.); chalika.w@chula.ac.th (C.W.); 3 Department of Orthopaedics, Faculty of Medicine, Chulalongkorn University, Bangkok 10330, Thailand; 4 Department of Veterinary Surgery, Faculty of Veterinary Science, Chulalongkorn University, Bangkok 10330, Thailand; 5 BIOtech Research Center, Department of Industrial Engineering, University of Trento, 38123 Trento, Italy; antonella.motta@unitn.it

**Keywords:** calcium–ortho-phosphate, clindamycin phosphate, amikacin sulfate, controlled-release, hydroxyapatite, beta-tricalcium phosphate, Thai silk fibroin, bone substitute

## Abstract

The porous particles prepared from composited calcium–ortho-phosphate (biphasic), Thai silk fibroin, gelatin, and alginate, with an organic to inorganic component ratio of 15.5:84.5, were tested for their abilities to control the release of the commercialized antibiotic solutions, clindamycin phosphate (CDP) and amikacin sulfate (AMK). The in vitro biodegradability tests complying to the ISO 10993-13:2010 standard showed that the particles degraded <20 wt% within 56 days. The drugs were loaded through a simple adsorption, with the maximum loading of injection-graded drug solution of 43.41 wt% for CDP, and 39.08 wt% for AMK. The release profiles from dissolution tests of the drug-loaded particles varied based on the adsorption methods used. The drug-loaded particles (without a drying step) released the drug immediately, while the drying process after the drug loading resulted in the sustained-release capability of the particles. The model-fitting of drug release profiles showed the release driven by diffusion with the first-ordered kinetic after the initial burst release. The released CDF and AMK from particles could sustain the inhibition of Gram-positive bacteria and Gram-negative bacteria, respectively, for at least 72 h. These results indicated the potential of these composited particles as controlled-release carriers for CDP and AMK.

## 1. Introduction

Challenges in treating bacterial bone infections that might occur in orthopedic surgery have been the topic of interest for decades. These post-operative infections and osteomyelitis can cause significant problems for both patients and the healthcare system. Traditional methods relying on systemic antibiotics require a high dose and long period of treatment with an increased risk of side effects and may not be effective to eradicate the infection. To address these limitations, controlled release systems have been developed to gradually deliver antibiotics directly to the infected site over an extended period, ensuring consistent therapeutic levels, reducing dosing frequency, and minimizing potential side effects [1]. Many broad-spectrum antibiotics have been used for local delivery, including Gentamycin, Amikacin, Vancomycin, and Clindamycin [2]. Commercially available bone cement products, Copal^®^, Palacos^®^, and Septopal^®^, primarily made of polymethylmethacrylate (PMMA) have been shown to be effective carriers for these antibiotic delivery systems [3,4]. The release kinetics of Vancomycin and Gentamicin from PMMA bone cements was suggested to be the combination of surface properties and porosity, showing initially burst-sustained release [5]. However, due to the non-degradability of PMMA, a second surgery is required for the removal of PMMA matrix. Biomaterials loaded with antibiotics for controlled release, which degrade during drug release, could then be an effective approach. Such biomaterials include several potential low- to non-toxic natural biomaterials such as silk fibroin, gelatin, and alginate.

Silk fibroin (SF), a natural fibrous protein from *Bombyx mori* silk cocoon, is known for its unique properties such as excellent mechanical strength, outstanding thermostability, as well as biocompatibility and biodegradability [6]. SF material, approved by the US Food and Drug Administration as a biomedical material [7], possesses adjustable biodegradability by tuning its blending, compositing, and crosslinking [8,9,10]. SF-based biomaterials also show negligible immunogenic reactions [7,8,11]. It was also applied as an organic polymer for controlled drug delivery systems [6,9,10]. Gelatin, partially hydrolyzed collagen, is another excellent biopolymer well-known for its biocompatibility and RGD sequence favorable for cell recruitment, resulting in numerous interests in related biomedical applications such as tissue-engineered scaffolds [12]. Alginate, a natural anionic polysaccharide, has been widely utilized as a carrier for drug delivery due to its mild gel formation properties at room temperature and in aqueous medium, competitive cost and source, biocompatibility, and FDA approval. As a result, various sensitive drugs, proteins, living cells, as well as enzymes have been successfully encapsulated in and released from alginate beads [13].

Calcium phosphate-based bone graft materials, including hydroxyapatite (HAp) and beta-tricalcium phosphate (*β*-TCP), have been utilized as therapeutic carriers for the management of bone diseases and injuries [14]. For example, the nanocrystalline HAp and calcium phosphate have been employed as carriers for the local administration of vancomycin and gentamicin [15]. Composite material systems comprised of various biodegradable matrix and calcium phosphate ceramics have been reported for their advantages in the controlled release of antibiotics. For example, composite material combining TCP with collagen (Cerasorb^®^ Foam, commercially available bone graft substitutes) was shown to enhance the drug loading and release of antibiotics (vancomycin and gentamicin) and provide more consistent antibiotic release compared to TCP (Cerasorb^®^ M) and HAp (Osbone^®^) granules [16].

In contrast to vancomycin and gentamicin, there are limited reports on the controlled release of Clindamycin- or Amikacin-loaded scaffolds prepared from natural polymers and ceramics composites. Clindamycin is a member of the linosamide group, inhibiting bacterial protein synthesis via the inhibition of bacterial ribosome at the 50S ribosome subunit. Clindamycin is primarily used for Gram-positive or anaerobic bacterial infections involving the skin, respiratory tract, bone infection, and oral cavity [17]. The most extensively studied drug delivery format for Clindamycin was particle-based, including both nano- and micro-sized particles. The method of emulsification was the most commonly used technique to prepare these particle-based drug delivery systems [4,18]. Other delivery formats such as membranes/films [19], three-dimensional structures [20], and fibers [21] can be found. The main approach for incorporating antibiotics into these materials involved directly mixing the antibiotics with solvent and materials, which is a convenient method. Various materials, both organic and inorganic, were used [4]. Organic polymers, in particular, alginate [17,19,22], fibroin [21], chitosan [19,23], and gelatin [22], received significant attention for drug delivery systems. Furthermore, organic–inorganic hybrid materials, such as calcium phosphate composites, were used to create Clindamycin delivery systems for treating bone infections. These drug delivery systems were found to be non-toxic to cells and could effectively inhibit bacterial proliferation and biofilm formation [18,23]. The inclusion of calcium phosphate components is advantageous because they are in bone composition, aiding cell proliferation, repair, and controlled drug release [18,23].

Amikacin is an aminoglycoside antibiotic, extensively used to treat infections that are resistant to other aminoglycosides, especially of those caused by Gram-negative bacteria. However, its limitations include a narrow therapeutic index and potential side effects on kidneys and ears. Additionally, it has low oral absorption [24]. Therefore, the delivery systems to address these limitations of Amikacin are required. Like Clindamycin, both natural and synthetic polymers have been used for developing Amikacin delivery systems [4,25]. Natural polymers, such as alginate [25], chitosan [26], and gelatin [27], have found wide application in Amikacin drug delivery systems. The most common technique used for preparing Amikacin nanoparticle-based systems is the solvent evaporation method, including water-in-oil (W/O) and oil-in-water (O/W) emulsification methods [24]. These Amikacin delivery systems have shown biocompatibility and minimal cytotoxicity in both in vitro and in vivo experiments [24].

Generally, porous particles of calcium ortho-phosphate or their composites are commonly reported as being used for bone substitutes [14]. In this work, the multifunctional bone regenerative particles made from composited calcium–ortho-phosphate porous and various organic compounds including alginate, SF, and gelatin, was developed and characterized. Its applicability as a carrier for the local delivery of antibiotics was investigated. Two antibiotics, Clindamycin and Amikacin, having different chemical structures, were selected. The encapsulation methods, encapsulation efficiency, drug-loading capacity, absorption rate, and in vitro drug release of the composited calcium–ortho-phosphate porous particle was investigated and compared.

## 2. Materials and Methods

### 2.1. Materials

Native Thai domestic silk cocoons (*Bombyx mori*), raced “Nangnoi Srisaket-1” were obtained from Queen Sirikit Sericulture Center, Nakhon Ratchasima province, Thailand. Type A gelatin from Nitta Gelatin Co., Osaka, Japan, sodium alginate medium viscosity, HAp, and *β*-TCP from Sigma-Aldrich, Steinheim, Germany, and dialysis membrane (MWCO 12,000–16,000 Da) from Viskase Company Inc., Tokyo, Japan, were used as received. Commercial antibiotics for injection, Clindamycin phosphate (CDP; Rosil, Bangkok, Thailand, 150 mg/mL), and Amikacin sulfate (AMK; The Government Pharmaceutical Organization, Bangkok, Thailand, 250 mg/2 mL), were used. Other chemicals used were analytical graded, if not specified otherwise, including sodium carbonate (Na_2_CO_3_; Ajax Finechem Pty., Ltd., Sydney, Australia), lithium bromide (LiBr; Sigma-Aldrich Laborchemikalien, Seelze, Germany), sodium azide (NaN_3_; Labchem, APS, Blacktown, Australia), TRIS hydrochloride (TRIS-HCL; 99%, Himedia™, Maharashtra, India), phosphate buffer (PBS; Bio basic Inc., Markham, ON, Canada), calcium chloride (CaCl_2_; USP testing specifications, Sigma-Aldrich, Germany), glutaraldehyde (Fluka, Neu-Ulm, Germany), and Collagenase type I (163 U/mg, GIBCO, Norristown, PA, USA).

### 2.2. Fabrication of Composited Calcium–Ortho-Phosphate Porous Particles

SF solution was prepared as reported previously [11]. In brief, silk gum was removed by boiling the cocoons in 0.02 M Na_2_CO_3_ solution. The obtained SF fiber was dissolved in 9.3 M LiBr solution at 60 °C for 4 h. The solution was dialyzed against deionized water using a dialysis membrane for 48 h. Alginate solution and gelatin solutions at the desired concentration were prepared in deionized water under continuous stirring until uniformity.

Composited calcium–ortho-phosphate porous particles were fabricated through the dispersion of inorganic powder biopolymer solutions and ionic crosslinking between sodium alginate and calcium chloride. The process involved dispersing a mixture of *β*-TCP and HAp (at 70% total wt./wt.) in DI water, followed by the addition of alginate, gelatin, and SF (at 30% total wt./wt.) solutions with continuous stirring until uniform. The mixture was then dropped into a CaCl_2_ solution, stirred for 15 min, and crosslinked with glutaraldehyde. After each step, the particulates were rinsed with DI water to remove excess chemicals. Finally, the obtained particulates were frozen, freeze-dried, and sent to be sterilized with gamma radiation (Thailand Institute of Nuclear Technology (Public Organization, Nakhon Nayok, Thailand) and kept refrigerated for further use.

### 2.3. Characterization of Composited Calcium–Ortho-Phosphate Porous Particle

The morphology and microstructure of composited calcium–ortho-phosphate porous particles was examined using scanning electron microscopy (SEM, JEOL JSM 5410LV, Tokyo, Japan). The particle size was analyzed using ImageJ 1.53q software (*n* = 50). Thermogravimetric analysis (TGA, Perkin Elmer’s model SII Diamond, Waltham, MA, USA) with a heating rate of 10 °C/min under a nitrogen atmosphere were employed to analyze the organic and inorganic compositions of particles. The absorption capacity of obtained particles was determined by immersing particles in phosphate buffer solution (PBS, pH 7.4) for 6 h at room temperature. The absorption capacity was calculated based on the weight of the particles before and after immersion. Total surface areas were measured by nitrogen adsorption using the Brunauer, Emmett, Teller (BET) method using a Quantachrome Instrument (Version 5.21, Quantachrome^®^ ASiQwin™–Automated Gas Sorption Data Acquisition and Reduction© 1994–2017, Vienna, Austria). The chemical structure of the particles before and after antibiotic adsorption was analyzed using Fourier Transform Infrared Spectroscopy (FTIR) in the wavelength range of 4000–400 cm^−1^ with an Attenuated Total Reflectance Fourier-Transform Infrared Spectroscopy (ATR-FTIR) mode on a Spectrum GX spectrometer (PerkinElmer, London, UK).

### 2.4. In Vitro Degradation

The study on the degradability of composited calcium–ortho-phosphate porous particles was conducted according to the requirements of the ISO 10993 standard for the biological evaluation of medical devices to identify and quantify potential degradation products. Due to the composited nature of the particles, the degradation testing followed both ISO 10993-13:2010 [28] for polymeric and ISO 10993-14:2001 [29] for ceramic medical devices. Detailed testing methods for both standards were described as follows.

The degradation testing for medical devices with polymeric components according to ISO 10993-13 [28] involves a real-time test in a simulated environment that closely resembles the in vivo conditions where the particles will be used. Phosphate buffer solution with collagenase IA enzyme at a concentration of 1 unit per milliliter (pH 7.4) was used. The particle samples were immersed in the solution, containing 0.01 wt% NaN_3_ to prevent bacterial growth. The samples are incubated at 37 °C for 56 days, with solution samples collected at intervals of 2, 4, 8, 14, 28, and 56 days. To ensure consistent contact between the test samples and the solution, 25% of the solution is replaced every 2 days. Samples immersed in the buffer solution without the enzyme was served as the control group. At each interval, the samples were centrifuged at 9000 rpm to separate the undigested solid particles from the clear solution. The degradability of the particles was assessed by measuring the percentage of remaining dry weight of solid and the amount of degradation products in clear solution (total protein content) using the Kjeldahl method [28].

The degradation testing for ceramic medical devices follows ISO 10993-14 [29], using a simulation test in a TRIS-HCI buffer solution (pH 7.4). The test began by immersing 0.5 g of particle samples in 10 mL of TRIS-HCI buffer solution. The samples were then incubated at 37 °C for 56 days while being shaken at a frequency of 2 Hz. Samples were collected at intervals of 2, 4, 8, 14, 28, and 56 days, with 25% of the solution being replaced every 2 days. The samples were then filtered through filter paper and then dried at 100 °C for 24 h until a constant weight was achieved. Similar to the previous test, the percentage of remaining dry weight was measured. The amount of degradation products which were calcium (Ca) and phosphorus (P) contents, in the filtered solution were also analyzed using Inductively Coupled Plasma Optical Emission Spectroscopy (ICP-OES, Thermo Scientific, Waltham, MA, USA).

### 2.5. Drug Loading

To process the drug loading into the particles, two commercially injectable antibiotics, including CDP at 600 mg per 4 mL (Rosil, Thailand) and AMK at 250 mg per 2 mL (Government Pharmaceutical Organization, Thailand) were used. Both drugs were diluted in phosphate-buffered saline to the desired concentration. Then, 150 microliters of the drug solution were carefully added to 25 mg of the particles. The mixture was incubated at 25 °C and shaken at 150 rpm for 30 min to ensure maximum adsorption. Afterward, the unabsorbed drug solution was removed.

Drug adsorption was tested using three different methods to assess the impact of different possible drug-loading methods on the drug release.

Method 1: The particles were immersed in drug solution and were immediately tested without drying.

Method 2: The particles were immersed in drug solution and were dried at 25 °C for 24 h before release testing.

Method 3: The particles were immersed in drug solution and were dried at 60 °C for 2 h before release testing.

To assess the drug-loading capacity and encapsulation efficiency of the particles, the Clindamycin-loaded and Amikacin-loaded particles were dissolved in a 1% *w/v* trisodium citrate solution. The mixture was vortexed and shaken at 150 rpm for 1 h. Afterward, the mixture was centrifuged at 9000 rpm for 10 min, and the supernatant was collected for drug-loading efficiency determination. Using a UV-visible spectrophotometer (Jenway, 6705 UV-vis, Stone, UK), the absorbance of the Clindamycin and Amikacin supernatants was measured at 207 nm and 335 nm, respectively. Light absorption of the blank (the solution with all components including the neat particles) was subtracted from the measured values for all experiments. The percentages of drug loading and encapsulation efficiency were calculated using the following equations (Equations (1) and (2)).


(1) $$\mathrm{Drug}\;\mathrm{loading}\;(\%\;\mathrm{DL})=\frac{\mathrm{Encapsulated}\;\mathrm{drug}\;(\mathrm{mg})}{\mathrm{Encapsulated}\;\mathrm{drug}+\mathrm{weight}\;\mathrm{of}\;\mathrm{particles}\;(\mathrm{mg})}\times 100$$

(2) $$\mathrm{Encapsulation}\;\mathrm{efficiency}\;(\%\;\mathrm{EE})\;=\;\frac{\mathrm{Encapsulated}\;\mathrm{drug}\;(\mathrm{mg})}{\mathrm{Total}\;\mathrm{drug}\;\mathrm{added}\;(\mathrm{mg})}\times 100$$

### 2.6. In Vitro Drug Release

An in vitro drug release study was conducted using a modified USP apparatus II (paddle method), with a phosphate buffer solution at pH 7.4 as the dissolution medium. Twenty-five (25) milligrams of drug-loaded particles (comparing different drug-loading methods 1, 2, and 3 and different drug concentrations) were added into a vessel containing 150 mL of phosphate buffer solution at 37 °C and the paddle’s rotation speed of 50 rpm. At specified time intervals, 1 mL of the dissolution medium was withdrawn and filtered through a nylon membrane (Filter Bio, Nantong, China) with a pore diameter of 0.22 µm. After each sample withdrawal, an equivalent volume of fresh solution was added. The concentrations of each released drug were determined using spectrophotometry, as described previously.

### 2.7. Antibacterial Activity of the Released Drug from the Particles

The study assessed the efficiency of the antibiotics’ activity of the released drug on the inhibition of bacterial growth using the modified disc diffusion method, following the standards of the Clinical and Laboratory Standards Institute (CLSI) [30]. The analysis involved testing on standard bacterial strains, including *Staphylococcus aureus* (*S. aureus*) ATCC 25923 (Gram-positive) and *Escherichia coli* (*E. coli*) ATCC 25922 (Gram-negative). The particles used for testing included those that absorbed CDP with Gram-positive bacteria and those that absorbed AMK with Gram-negative bacteria. The control group consisted of neat particles, without drug loading.

The testing process began with culturing bacteria on cell culture media at 37 °C for 24 h. Subsequently, 5 milligrams of the composited calcium–ortho-phosphate particles were absorbed with antibiotics and dried at 25 °C for 24 h. These particles were then placed on the surface of agar plates for bacterial culture. The plates were incubated at 37 °C with 90% humidity in a dark environment. The size of clear zones around the particles was measured at 24, 48, and 72 h.

### 2.8. Statistical Analysis

The comparison of data was conducted by collecting three replicates (*n* = 3) and presenting them as the average and standard deviation (SD) to analyze statistically significant differences. Analysis of Variance (ANOVA) was employed using IBM SPSS Statistics 22 software at a confidence level of 95% (*p*-value < 0.05).

## 3. Results and Discussion

### 3.1. Characteristics of Composited Calcium–Ortho-Phosphate Porous Particles

**Figure 1:**
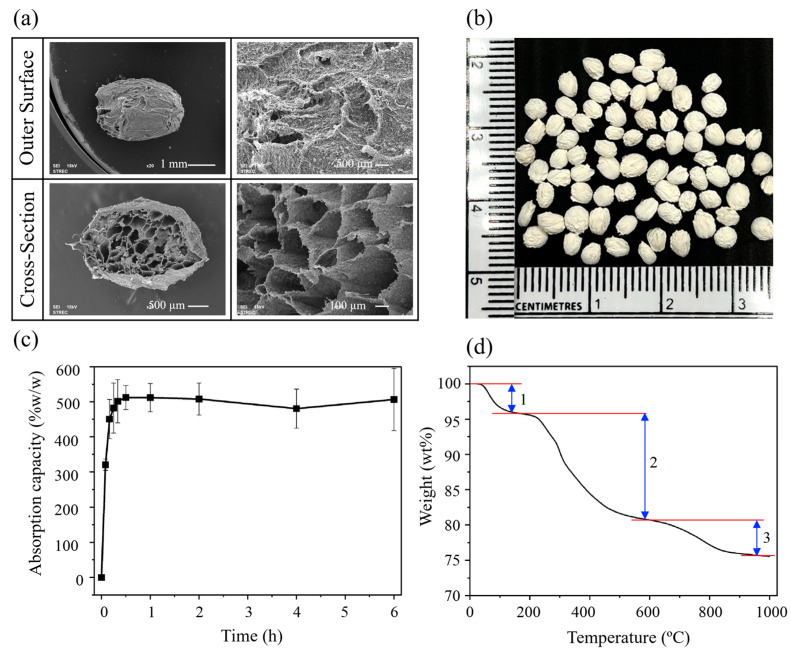
(**a**) SEM images of particles (outer and cross-sectional surfaces), (**b**) appearance of the particles, (**c**) absorption capacity of particles in PBS, pH 7.4 at 25 °C, (values reported are an average (*n* = 3) ± standard deviation), (**d**) TGA thermogram of particles at the heating rate of 10 °C/min). Numbers 1–3 referred to three main states of weight loss.

The composited calcium–ortho-phosphate porous particles formed when a droplet of mixed solution of natural polymers and calcium phosphate was in contact with CaCl_2_ solution, where the alginate was able to form a three-dimensional network through ionic crosslinking with divalent cations (Ca^2+^). The calcium ions could diffuse into the gel matrix, ionic bonded with the alginate molecules and forming a structure of an “egg box model” [13]. As known, calcium ions diffused into the gaps in the alginate chains, binding the alginate molecules and forming a structure resembling an egg box model. Figure 1a,b show the SEM morphology and physical appearance of the formed particles. The fabricated particles were elliptical and spherical in shape with a rough surface. The surface roughness has been caused by *β*-TCP and HAp powders. Moreover, the particles exhibited wrinkles on their surfaces due to the freeze-drying process [31,32].

The fabricated particles had an average width diameter of 2.78 ± 0.10 mm, and the long-sided diameter of 3.19 ± 0.08 mm. The cross-sectional morphology of these particles revealed a porous structure with interconnected pores. The pore sizes ranged from 100 to 900 µm, with an average size of 364 ± 72 µm, porosity of 69.36 ± 5.23%, and particle density of 0.34 ± 0.02 g/cm^3^. Furthermore, the density of the particles was 0.0160 ± 0.01 g/cm^3^ while the total surface area (BET) was 23.73m^2^/g (Table 1).

The absorption capacity of the particles evaluated in PBS (pH 7.4) is illustrated in Figure 1c. The particles exhibited the saturated liquid absorption of 500 wt% after 6 h. A rapid uptake of PBS was observed within the initial 15 min. However, after the first 30 min, no further absorption capacity was observed, signifying that the saturation point occurred within this half-hour. This outcome is attributed to the inherent hydrophilic properties of gelatin and alginate, which are the components of the particles [33,34]. The liquid absorption is a crucial characteristic in the evaluation of biomaterials employed as carriers and scaffolds in tissue engineering. It is necessary for the absorption of body fluid, which is primarily composed of water, as well as the transfer of cell nutrients and metabolites [35]. A summary of the physical characterization of the particles is shown in Table 1.

The composition of the particles was determined using thermogravimetric analysis (TGA). Figure 1d illustrates the change in mass percentage of particles upon heating. The mass loss percentage curve showed three main stages of weight loss: Initially, there was a weight loss below 100 °C due to water evaporation, approximately 3.90 wt%. The second stage involved the degradation of natural organic polymers, namely SF, gelatin, and alginate, occurring within the temperature range of 180–450 °C [36]. The thermal decomposition of SF and gelatin began at approximately 200 °C [37] and calcium alginate began at approximately 250 °C [38]. In the third stage, taking place at 600–800 °C, the decomposition of calcium carbonate compounds occurred through a carboxylic group elimination reaction (-COOH) or decarboxylation. This process resulted in the release of carbon dioxide and subsequent weight loss [38].

This revealed that the weight ratio of organic to inorganic components in the particles was 15.46 to 84.54. Noted that the organic to inorganic ratio from the initial input at fabrication fraction was 30:70. This change in the composition ratios was mainly a result of organic material loss during the production processes such as washing steps.

### 3.2. In Vitro Degradation of Composited Calcium–Ortho-Phosphate Porous Particle

**Figure 2:**
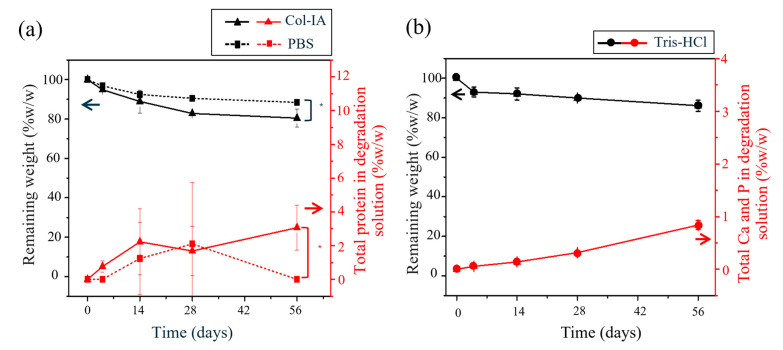
(**a**) Remaining weight of particles and total soluble protein in two degradation solutions, col IA (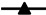,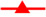) and PBS (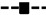,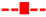), at pH 7.4 and 37 °C, according to ISO 10993-13. (**b**) Remaining weight of particles and total calcium (Ca) and phosphorus (P) in degradation solution (TRIS-HCI buffer solution 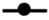,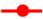) at pH 7.4 and 37 °C, according to ISO 10993-14. Values reported are an average ± standard deviation (*n* = 3) and * indicates statistically significant differences at *p* < 0.05 between two degradation solutions at 56 days.

Degradation results of the organic contents of the particles, evaluated according to the ISO 10993-13:2010, using PBS with collagenase IA enzyme (col IA) and without enzyme at pH 7.4 and 37 °C were shown in Figure 2a. It was observed that after 4 days, the particles immersed in PBS with col IA began to fragment into smaller pieces, eventually losing their original shape entirely by day 14. In contrast, the particles immersed in PBS commenced fragmenting after 8 days, with complete deformation occurring by day 56. The degradation process in both solutions was characterized as bulk degradation. It was observed that the degradation process in the presence of col IA led to lower percentages of the remaining weight in comparison to PBS. At 56 days, the particles immersed in col IA and PBS exhibited remaining weight percentages of 80.46 ± 4.63 and 88.47 ± 0.69, respectively. The decrease in the remaining weight percentage of the particles during the testing period is associated with a continuous rise in the total soluble protein content in the degradation product solution. The total soluble protein in the degradation solution was greater in the presence of col IA compared to PBS. This increase in total soluble proteins in the presence of col IA was driven by the enzymatic cleavage of peptide bonds in gelatin and SF [39]. Hydrolysis reactions played a pivotal role in these mechanisms. The degradation process was catalyzed by collagenase enzymes, with a particular impact on gelatin. Additionally, collagenase could break peptide bonds in SF chains. They targeted peptide bonds between glycine and amino acid X in the amino acid sequence (X-Gly-Pro)_n_. The primary amino acids represented by X include lysine, arginine, methionine, and valine [40]. Consequently, this degradation process led to molecular weight alterations in gelatin and fibroin, resulting in reduced entanglement of polymer chains. Furthermore, the alginate within the particles was in the form of calcium alginate and, exposed to a PBS, it underwent a transition into sodium alginate. This transformation resulted from an ion exchange process in which calcium ions were replaced by sodium ions. This subsequent alteration enabled degradation through hydrolysis, involving the cleavage of glycosidic bonds that linked alginate monomers and ultimately leading to the disintegration of polysaccharide chains [41].

The degradation of inorganic contents of the particles was evaluated according to the ISO 10993-14, a standard designed for ceramic medical devices (in this case, calcium–ortho-phosphates) using Tris-HCl buffer solution at pH 7.4 and 37 °C. The findings, shown in Figure 2b, revealed that after eight days, the particles initiated the process of fragmenting into smaller pieces. The percentage of remaining weight consistently decreased as the duration of the testing period increased, ultimately reaching 86.17 ± 2.83 at 56 days. This closely aligned with the percentages obtained in a PBS, as detailed in a polymeric medical device test. The weight loss of the particles could be attributed to the degradation of organic components such as SF, gelatin, and alginate, as well as the dissolution of the inorganic components, including calcium phosphate. The percentage of remaining weight aligned with the increase of total calcium and phosphorus in the degradation product solution. The total calcium and phosphorus content in the analyzed degradation product solution was relatively low, less than 0.9 wt%. This may be due to the slow degradation of calcium phosphate compounds and the enhanced stability of the particles through improvements in their physical and chemical properties. In vitro biodegradation of particles involves a combination of physical and chemical degradation processes, physical processes such as erosion and particle fragmentation, as well as chemical processes involving dissolution [42]. The dissolution of calcium phosphate compounds is influenced by various factors, such as solubility, the ratio of calcium phosphate, pH, fluid convection, and temperature [42]. *β*-TCP exhibits greater solubility than HAp in environments with a pH around 7. However, both calcium phosphate compounds exhibit relatively low solubility, resulting in the slow degradation of particles [43]. As demonstrated by He, F. et al. [44], degradation of materials with a 55:45 weight ratio of *β*-TCP and HAp (similar to the composition of the particles in our study) exhibited a weight loss of 0.44 wt% after 56 days of immersion in a Tris-HCl solution.

### 3.3. Drug Loading and Entrapment Efficiency of the Particles

**Figure 3:**
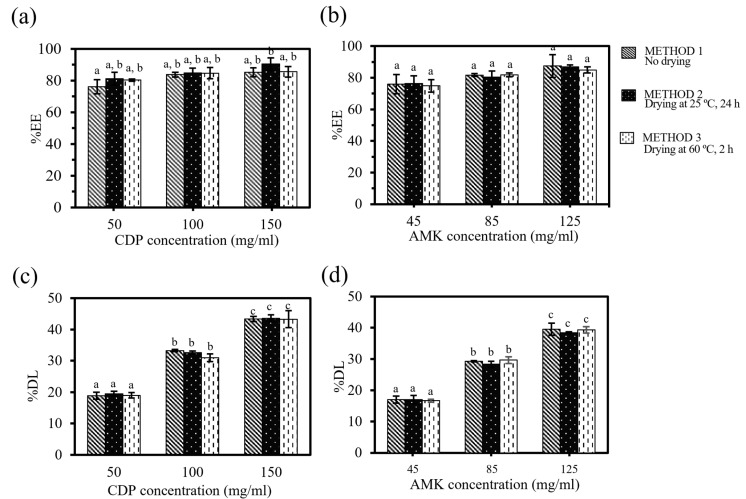
Encapsulation efficiency (% EE) (**a**,**b**) and drug-loading capacity (% DL), (**c**,**d**) for CDP (**a**,**c**) and AMK (**b**,**d**) particles with various absorption methods (Method 1, Method 2, Method 3). The letter a–c indicate statistically significant differences at *p* < 0.05. for each set of data.

Drug loading and entrapment efficiency are crucial factors in assessing the suitability of carriers for drug delivery systems [45]. The ability of particles to adsorb drugs depends on the physical and chemical properties of carrier materials, including polarity, solubility, and surface area [46]. As shown in Figure 3a,b, the encapsulation efficiency (% EE) of CDP and AMK on particles tended to increase when the concentration of the drug was increased. The highest encapsulation efficiencies were 87.03 ± 2.90% for CDP and 86.44 ± 1.32% for AMK, at the concentration of each commercially available drug (highest concentration of each drug). When considering the impacts of the three different drug adsorption methods on the entrapment efficiency, it was found that the entrapment efficiency percentages were similar across the absorption methods regardless of drying or no-drying applied to the drug-immersed particles.

For drug-loading capacity shown in Figure 3c,d, it depended only on the concentration of the antibiotic drug, but not the adsorption methods involving non-drying or drying of the drug-immersed particles. The percentage of the maximum amount of drug adsorbed on the particles for CDP at the concentration of 150 mg/mL was 43.41 ± 0.15, while the drug-loading capacity of AMK at the concentration of 125 mg/mL entrapped in the particles was 39.08 ± 1.22. The drug concentration has a significant impact on the drug-loading capacity of the particle whereas there is no significant difference in encapsulation efficiency. This phenomenon occurs because the process of drug-loaded particles relies on adsorption, which is a process where the drug molecules (adsorbate) accumulate on the surface of a carrier (adsorbent). Consequently, the rate of adsorption exhibits a direct correlation with the concentration of the drug, assuming the free surface area of the adsorbent remains constant [47].

Three different absorption methods of drug solutions into the porous particles were utilized to mimic the possible methods of application by either manufacturing or users. The results showed that for a general method of immersing the particles in the drug solution with or without drying, the drying steps had no significant effects on drug-loading capacity and entrapment efficiency. The drying processes at the temperature of 24 or 60 °C only involve the removal of water molecules at the temperatures lower than the decomposition temperatures of the antibiotics (172.4 °C of CDP [48] and 200 °C of AMK [24]).

**Figure 4:**
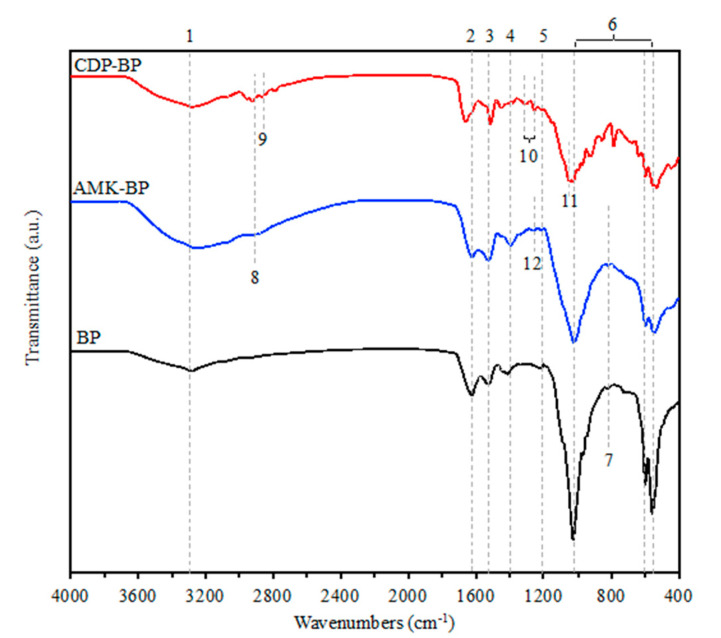
FTIR spectra of Clindamycin-loaded particle (CDP-BP), Amikacin-loaded particle (AMK-BP), and particle without drug loading (BP). Numbers on the figure corresponded to the peak numbers in Table 2.

FTIR spectroscopy results shown in Figure 4 and Table 2 were employed to confirm the incorporation of CDP and AMK into the particles. The spectrum of neat particles without drug loading (BP) showed the vibration of the O-H stretching at 3278 cm^−1^, possibly overlapping with the N-H stretching. This indicates hydrogen bond formation due to intermolecular interactions between silk fibroin, alginate, and gelatin molecules [39]. The peaks at 1624 cm^−1^ (C=O stretching) and 1524 cm^−1^ (N-H bending) correspond to amide I and amide II, respectively, indicating *β*-sheet structure of fibroin [49]. The peak at 1214 cm^−1^ represents an amide III from the vibration of the C-N bond, suggesting a random coil or α-helix structure. Amides I, II, and III indicate the proteins that constitute the particle. Additionally, the peak at 1416 cm^−1^ corresponds to the symmetric stretching of carboxylate salt, presenting carbohydrate, specifically alginate, in the particles [33]. Peaks at 559, 599, and 1026 cm^−1^ correspond to the phosphate group (PO_4_^3−^) in calcium phosphate compounds [50]. At 725 cm^−1^, the peak indicates the presence of the pyrophosphate group (P_2_O_7_^4−^) found exclusively in β-TCP [50,51]. After the incorporation of CDP into the particles, the FTIR spectrum of the CDP-loaded particles (CDP-BP) showed -CH stretching vibration peaks were visible at 2955 and 2923 cm^−1^, while that for S-C-H appeared at 1255 and 1208 cm^−1^. In addition, the CDP-BP exhibited a peak at 1046 cm^−1^ representing the C-N stretching. These results confirmed the presence of CDP in the particles. The primary structural components of Clindamycin molecules, characterized by pyrrole and saccharide ring vibrations, displayed skeletal vibrations between 1600 and 600 cm^−1^ [52]. The bands in this region were primarily associated with C double bond tensile vibrations. Significant changes were noted at approximately 1047 cm^−1^, corresponding to C-C stretching in the pyrrolidine group. Observations from the FTIR analysis revealed that peaks corresponding to specific functional groups in the drug-loaded particles were slightly shifted when compared to particles without drug loading. This shift may indicate the occurrence of interactions between the drug and the matrix. In case of CDP-BP, one possible explanation is the formation of hydrogen bonds between the hydroxyl group (-OH) of CDP and carboxyl groups (-COOH) present in the structure of SF, gelatin, and alginate [53]. Additionally, electrostatic interactions could have occurred between the negatively charged oxygen atom in the CDP molecular structure and the positively charged surface of calcium phosphate (specifically, *β*-TCP and HAp) at the site of calcium ions (Ca^2+^) and phosphate group (${\mathrm{PO}}_{4}^{3-}$) [54].

Considering Amikacin-loaded particles, the presence of oxygen atoms in the AMK molecule allows for potential electrostatic interactions with positively charged calcium ions (Ca^2+^) on the surface of calcium phosphate. Additionally, positively charged nitrogen atoms in AMK can interact with negatively charged surface groups on the phosphate group (${\mathrm{PO}}_{4}^{3-}$). Hydrogen bonds may form between the amino groups (-NH_2_) and hydroxyl groups (-OH) in the molecular structure of Amikacin sulfate. These bonds can potentially interact with the hydroxyl groups (-OH) present in SF, gelatin, and alginate. Furthermore, it was observed that the particles that have undergone absorption of AMK exhibited a noticeable shift in peak position number 4 (Figure 4), which may be the result of interactions between the amino groups (-NH_2_) of AMK and the carboxyl groups (-COOH) in the alginate [55]. In addition, the availability of negatively charged carboxyl groups in the SF may also interact with positively charged ammonium groups in AMK.

The diverse biomaterial composition of particles, including SF, gelatin, alginate, *β*-TCP, and HAp, enables varied interactions with drug molecules. FTIR analysis revealed no new chemical bonds formed after drug absorption, indicating a physical adsorption process involving intermolecular interactions like electrostatic interactions and possibly hydrogen bonds between drug molecules and the particles.

### 3.4. In Vitro Drug Release of CDP or AMK from the Particles

**Figure 5:**
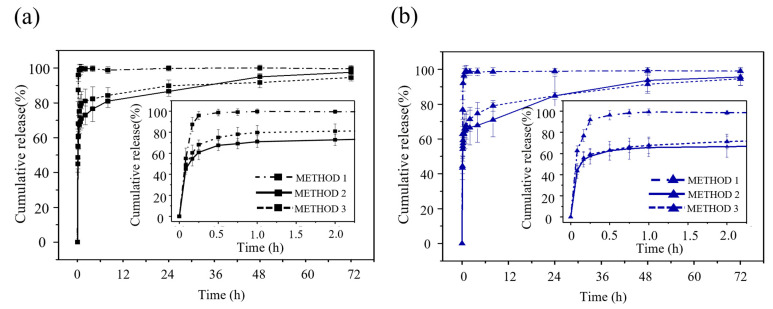
Cumulative release profiles of (**a**) CDP at 150 mg/mL and (**b**) AMK at 125 mg/mL from particles subjected to three different absorption methods in PBS, pH 7.4 at 37 °C. Values reported are an average ± standard deviation (*n* = 6).

**Figure 6:**
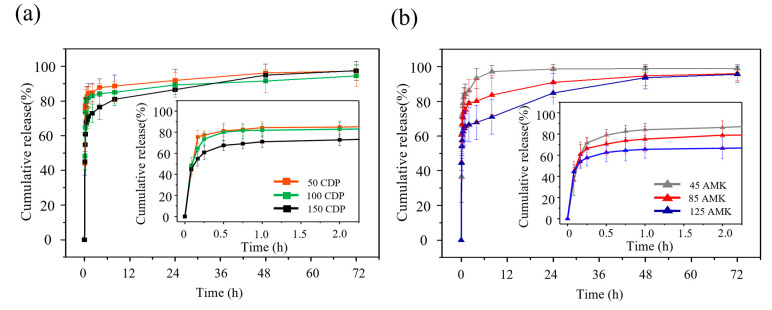
Cumulative release profiles of (**a**) CDP and (**b**) AMK from particles in PBS, pH 7.4 at 37 °C (drug loading method 2). Values reported are an average ± standard deviation (*n* = 6).

In vitro dissolution testing is a standard method to determine the profile and the amount of released drug from a solid form. Procedures must be standardized in order to evaluate the kinetic of dissolution and pattern of drug from the carriers. In this study, in vitro drug release tests were performed according to the USP pharmacopoeia dissolution test [56] in a modified paddle dissolution apparatus. The release profiles of two antibiotics, CDP and AMK with each highest drug concentration available, from the natural biomaterial-based particles subjected to different drug absorption methods and drug loading are illustrated in Figure 5 and Figure 6.

In Figure 5, the particles subjected to the adsorption method 1 (without drying) rapidly released both drugs, reaching a cumulative release of about 99% within just 45 min. This is classified as immediate release according to the FDA guidelines on “Dissolution Testing and Acceptance Criteria for Immediate-Release Solid Oral Dosage Form Drug Products Containing High Solubility Drug Substances” (at least 80% of the drug to dissolve within 30 min) [57]. For particles subjected to the adsorption method 2 and 3, which included drying steps at 25 °C for 24 h and 60 °C for 2 h, respectively, before the dissolution test, the initial burst releases of both drugs were presented in the first 15 min, with the cumulative release percentages around 60% for CDP and 57% for Amikacin sulfate. The cumulative release then gradually increased, reaching about 95% after 72 h. The release patterns for the particles with drug-loading method 2 and 3 were similar to each other and showed slower release behavior compared to those with drug-loading method 1. This indicated that for the absorption of the drug in a saturated solution, the drug molecules trended to have more interaction with the surrounding medium and weaker interaction to the surface of the porous particles. For the absorption method 2 and 3 with drying process, the rapid cumulative release can be observed in the first 15 min, then gradual releases were observed during 6–48 h although the total cumulative releases at 72 h were at a similar point for all samples and for both drug-carrier systems. These results emphasized the effects of the drying process upon drug loading on these particles which could increase the drug molecules–carrier surface interactions which could enhance their controlled release abilities.

The release profiles of two antibiotics at various drug concentrations from the dried particles (drug loading method 2) were presented in Figure 6. The release of CDP and AMK from particles could be divided into two main stages. Initially, within the first 15 min, the particles exhibited rapid drug release. Subsequently, the release slowly progressed, reaching a cumulative release of approximately 95–100 after 72 h. The particles with higher drug loading exhibited lower cumulative release percentages. This phenomenon could be attributed to increased molecular interactions among drug molecules at higher concentrations, leading to a reduced diffusion rate into the surrounding medium. Furthermore, several factors could also influence drug release, including drug solubility, surface area of carrier, solvent viscosity, pH of solvent, and test conditions such as temperature and agitation speed [58]. When drugs absorbed onto the particles through electrostatic and hydrogen bonding interactions were subjected to drug release testing in a phosphate buffer solution (pH 7.4) at 37 °C with continuous agitation, these interactions were disrupted. This disturbance led to the release of drugs from the particles, as hydrogen bonding interactions are known to weaken with increasing environmental temperature [59]. Similarly, mechanical forces such as shear and compression can dismantle electrostatic interactions [60].

In a comparison of two drugs, CDP and AMK, CDP showed a higher cumulative release rate than AMK. This might be caused by the high solubility of CDP in water (more than 150 mg/mL) [23] compared to that of AMK (50 mg/mL) [61]. Water solubility plays a critical role in determining diffusion rates of highly hydrophilic drugs. Additionally, highly hydrophilic drugs contribute to the overall hydrophilicity of the drug delivery system, promoting increased water absorption and faster drug release. Furthermore, the molecular weight of CDP (505 g/mol) which is lower than that of AMK (781 g/mol), could facilitate its diffusion from the particles. This phenomenon aligns with Fick’s First Law, which states that the diffusion coefficient of a substance is directly proportional to temperature and inversely proportional to liquid viscosity as well as the size of particles or molecules [62].

The results obtained were applied to evaluate the kinetics of CDP and AMK release from the particles. From the drug release profiles, it revealed that the release pattern can be segmented into two distinct stages: an initial burst release within the first 15 min, followed by a slow release extending up to 72 h. For this study, the mathematical modeling of the release profile of the drug was considered after the burst release section using zero order, first order, Higuchi’s, and Korsmeyer–Peppas release models [55,60,61]

(3) $$\mathrm{Zero order}:\;Q=\mathrm{kt}+Q_{0}\;$$

(4) $$\mathrm{First order}:\;Q=Q_{0}\;({1-e}^{-kt})$$

(5) $$\mathrm{Higuchi}’s:\;Q={\mathrm{kt}}^{1/2}\;$$

(6) $$\mathrm{Korsmeyer}–\mathrm{Peppas}:Q={\mathrm{kt}}^{n}\;$$

where Q is the amount/concentration of drug released at time t, Q_0_ is the initial amount/concentration of drug, k is the rate constant, t is time, and *n* indicates the mechanism of release (where *n* < 0.43 represents Fickian diffusion, and 0.43 < *n* < 0.85 is characteristic for anomalous transport).

Table 3 provides a summary of the determined correlation coefficients (R^2^) and the rate constants, k, obtained from different mathematical models. Based on the R^2^ values, the slow release observed after the initial burst release of both antibiotics appeared to conform to the mixing characteristic of the first order and the Korsmeyer–Peppas release models, with the “*n*” pointed to Fickian diffusion (*n* < 0.43) [58]. This finding elucidated the release of CDP and AMK from the particles and might be mainly from the diffusion mechanism, from the surfaces of the composited calcium–ortho-phosphate porous particles into the external medium solution, driven by the concentration gradient [63]. The particles composed mainly of the inorganic calcium–ortho-phosphates, around 85% (wt), glued together with blended organic materials of SF, gelatin, and alginate, where the hydrophilic properties of gelatin and alginate enable the particles to absorb water and undergo swelling. Consequently, the drug trapped within the particles was released throughout the particle matrix via diffusion. Furthermore, the biodegradability of natural polymers in the matrix could also enhance the drug release. Therefore, the dominant mechanism governing drug release is diffusion, a phenomenon that closely aligns with first-order release kinetics and the Korsmeyer–Peppas release model. The evidence of the in vitro degradation test of the carriers in Figure 2 show very low degradation in the first 72 h (<5%, with or without enzymes), indicating that the degradation of the carriers had minimal effects on the release profiles.

### 3.5. Antibacterial Activity of the Released Drug

The antibacterial activities were assessed using the drug-loaded particles (absorption method 2) at the highest doses (150 CDP-BP and 125 AMK-BP). The particles without drug loading were utilized as a control group. *S. aureus* (gram positive) and *E. coli* (gram negative) are the most common bacteria responsible for the infections. Table 4 showed the clear zones of drug-loaded particles against *S. aureus* for 150 CDP-BP and the inhibition of *E. coli* for 125 AMK-BP over time intervals. The results showed that drug-loaded particles effectively inhibited the growth of *S. aureus* and *E. coli*. The average clear zone diameters for different time intervals were not significantly different, indicating bacterial inhibition for up to 72 h. However, in this test, the burst release characteristic in both samples was not observed in the first 24 h due to the different medium of the drug transport. Burst diffusion of a drug is generally observed in solution but not in gel.

The effectiveness of bacterial inhibition is influenced by various factors, such as antibiotic diffusion rate, surrounding environment, and bacterial growth rate. While the clear zones observed in standard diffusion tests provide initial insights, they may not accurately predict antibacterial efficacy in complex in vivo conditions. In the treatment of bone defect which antibiotics must diffuse through tissues in multiple directions, a specialized drug delivery system could enhance drug concentration in infected areas and improve treatment outcomes. In addition, the cytotoxicity of our developed composited particle was certified according to the ISO 10993-5:2009 standard by The Department of Medical Science, Ministry of Public Health, Nontaburi, Thailand. This study demonstrates the potential of composited calcium–ortho-phosphate porous particles for the controlled release of CDP and AMK.

## 4. Conclusions

We showed the evaluation results of bone filler particles prepared from a composite of calcium–ortho-phosphate, Thai silk fibroin, gelatin, and alginate on their controlled-release function for two antibiotics, Clindamycin phosphate (CDP) and Amikacin sulfate (AMK). The antibiotic loading efficiency of the particles could be achieved using simple adsorption. The percentages of the maximum drug loading on the particles for injection graded-commercialized drug solution were at 43.41 ± 0.15 for CDP, and at 39.08 ± 1.22 for AMK. Different release profiles of the antibiotic-loaded particles depended on the adsorption methods. A dissolution test of the dried particles which absorbed the drug solution without a drying step exhibited immediate release, while the drug-loaded particles with a drying process showed a burst initial release followed by a sustained release pattern. Mathematical model fitting of the release profiles of both drugs indicated that their controlled-release characteristics were mainly the diffusion controlled with the first-ordered kinetic. The CDP released from the composite of calcium–ortho-phosphate effectively inhibited Gram-positive bacteria (*S. aureus*), while the AMK-loaded particles effectively inhibited Gram-negative bacteria (*E. coli*). The effectiveness of both drugs was shown to be sustained for at least 72 h by the stable clear zones of bacterial inhibition.

In vitro degradation tests of the blank particles in simulated body fluid conditions, complying to the biological evaluation standards for medical devices, for polymers (ISO 10993-13:2010) and ceramic (ISO 10993-14:2001) showed >80% remaining of the particles in 56 days. This highlights the potential application of these composited calcium–ortho-phosphate porous particles, as a multifunctional bone regeneration device with controlled-release capability of antibiotics for localized infection during an initial period of treatment. These particles will be further evaluated for use as bone substitute materials in orthopedic surgery.

## Figures and Tables

**Table 1:** Summary of physical characterization of composited calcium–ortho-phosphate porous particles.

Characteristics	Values	Method of Evaluation
Average sizes (*n* = 50)	Diameter (width) 2.78 ± 0.10 mm, (length) 3.19 ± 0.08 mm	SEM and Image J 1.53q software
Pore size (*n* = 50)	Macropores 100–900 µm Average size of 364 ± 72 µm	
Porosity (*n* = 50)	69.36 ± 5.23%	
Total surface areas	23.73 m^2^/g Micropore surface area 7.258 m^2^/g	BET
Density (*n* = 100)	0.34 ± 0.02 g/cm^3^	Average Volume/ Average Mass
Absorption	Approximately 500 times in PBS (pH 7.4, 37 °C) Within 30 min	-

**Table 2:** Comparison of FTIR peaks of Clindamycin-loaded particles (CDP-BP), Amikacin-loaded particles (AMK-BP), and particles without drug loading (BP).

Peak No.	Reference Wavenumber (cm^−1^)	Functional Group	Wavenumber (cm^−1^)		
			BP	CDP-BP	AMK-BP
1	3200–3600	O-H stretching	3278	3275	3271
2	1620–1690	amide I (C=O stretching)	1624	1660	1623
3	1481–1550	amide II (N-H bending)	1524	1513	1525
4	1395–1440	COO−	1416	1421	1395
5	1200–1400	amide III (C-N stretching)	1214	1208	1213
6	1020–1120 560–600	PO_4_^3−^	1026	1026	1022
			599	598	599
			559	553	551
7	720–730	P_2_O_7_^4−^	725		
8	2840–3000	C-H stretching	-	2955	-
9	2840–3000	C-H stretching	-	2923	2936
10	1209–1249	S-C-H stretching	-	1255	-
			-	1208	-
11	1020–1250	C-N (Stretching) (Aliphatic amine)	-	1046	-
12	1000–1260	C-O stretching	-	-	1264

**Table 3:** Summary of the release kinetic of CDP-BP and AMK-BP, from 15 min to 72 h, according to four drug release models (zero order, first order, Higuchi’s, and Korsmeyer–Peppas).

Sample	Zero Order		First Order		Higuchi		Korsmeyer–Peppas	
	k	R^2^	k	R^2^	k	R^2^	R^2^	*n*
	(mg/mL·h)		(h^−1^)		(mg/mL·h^1/2^)			
150 CDP	0.0752	0.77	0.0199	0.94	0.7008	0.87	0.96	0.29
100 CDP	0.0333	0.80	0.0221	0.96	0.3109	0.91	0.90	0.25
50 CDP	0.0133	0.77	0.0226	0.96	0.1258	0.90	0.97	0.33
125 AMK	0.0811	0.89	0.0251	0.99	0.7431	0.97	0.99	0.30
85 AMK	0.0343	0.75	0.0260	0.99	0.3288	0.90	0.93	0.19
45 AMK	0.0140	0.49	0.0270	0.76	0.1442	0.65	0.90	0.43

**Table 4:** Kinetics of the clear zone, representing antibacterial activity of CDP-BP- and AMK-BP-loaded composited calcium–ortho-phosphate porous particles. The values reported are an average ± standard deviation (*n* = 3) and different letters indicate statistically significant differences at *p* < 0.05 among the data set of each drug-loaded particle.

Time	Photographs and Diameter of Clear Zone (mm)	
	*S. aureus*	*E. coli*
	Control (Particle Only)	Control (Particle Only)
24 h	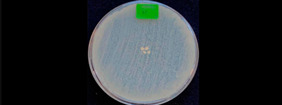	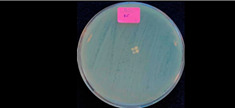
	0.00 ± 0.00 mm	0.00 ± 0.00 mm
	CDP-BP	AMK-BP
24 h	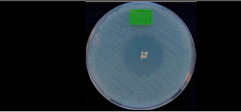	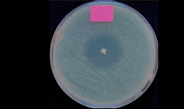
	25.67 ± 1.53 ^a^ mm	22.00 ± 0.00 ^b^ mm
48 h	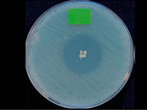	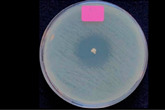
	25.00 ± 1.00 ^a^ mm	20.67 ± 1.15 ^b^ mm
72 h	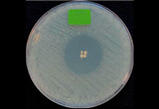	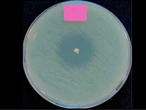
	24.67 ± 1.15 ^a^ mm	20.00 ± 0.00 ^b^ mm

## Data Availability

The original contributions presented in the study are included in the article, further inquiries can be directed to the corresponding author.
